# TTF-1、CD56、P40免疫组化标志物及其他临床特征与小细胞肺癌预后相关性研究

**DOI:** 10.3779/j.issn.1009-3419.2017.08.04

**Published:** 2017-08-20

**Authors:** 鑫 王, 毅 张, 牧 胡, 若天 王, 磊 刘, 坤 钱, 元博 李, 修益 支

**Affiliations:** 100053 北京，首都医科大学宣武医院胸外科 Department of Thoracic Surgery, Xuanwu Hospital, Capital Medical University, Beijing 100053, China

**Keywords:** 肺肿瘤, 预后, 甲状腺转录因子-1, Lung neoplsms, Prognosis, Thyroid transcription factor-1

## Abstract

**背景与目的:**

本研究旨在分析甲状腺转录因子-1（thyroid transcription factor-1, TTF-1）、神经细胞粘附分子CD56和P40蛋白在小细胞肺癌患者中的阳性表达情况，探讨上述免疫表型标志物及其他临床特征与小细胞肺癌预后的相关性。

**方法:**

用免疫组织化学方法检测198例初次诊治的小细胞肺癌患者石蜡包埋活检组织标本中TTF-1、CD56、P40的阳性表达情况，观察随访患者临床特征及治疗、生存情况，通过Cox风险比例模型分析上述标志物、临床病理特征与预后的相关性。

**结果:**

198例小细胞肺癌患者TTF-1、CD56、P40的阳性率分别为73.2%（145/198）、88.4%（175/198）、7.1%（14/198）。TTF-1是否阳性为小细胞肺癌患者独立预后因素OR=0.665，95%CI：0.472-0.937。其他与预后的相关因素包括：与不吸烟者相比，吸烟指数≤400组OR=1.72，95%CI：1.061-2.789；美国东部肿瘤协作组（Eastern Cooperative Oncology Group, ECOG）得分为2分与0分者相比的OR=3.551，95%CI：2.133-5.914；广泛期与局限期患者相比OR=2.487，95%CI：1.793-3.451；合并上腔静脉压迫综合征（superior vena cava syndrome, SVCS）者OR=2.394，95%CI：1.49-3.846。

**结论:**

小细胞肺癌中的预后与TTF-1表达及吸烟、ECOG得分、肿瘤分期、合并SVCS等多个因素相关，TTF-1、CD56、P40表达在小细胞肺癌的诊断和鉴别诊断中有辅助作用。

## Abstract (en)

**Background and objective:**

The aim of this study is to explore roles of thyroid transcription factor-1 (TTF-1), CD56, P40 expression and other clinical characteristics predicting response and survival in patients with small cell lung cancer (SCLC).

**Methods:**

Formalin-fixed, paraffin-embedded biopsy tissues were retrospectively obtained from 198 SCLC patients who were diagnosed first in Xuanwu Hospital. The expressions of TTF-1, CD56 and P40 were detected by immunohistochemistry. The clinical data including age, gender, cancer stage, Eastern Cooperative Oncology Group (ECOG) score, smoking or not, superior vena cava syndrome (SVCS) due to lung cancer or not were collected. Cox proportional hazard model was used to analyze the relationship between the overall survival (OS) and factors.

**Results:**

Immunohistochemical staining results showed the positive rate of TTF-1, CD56, P40 were 73.2%, 88.4% and 7.1% respectively. TTF-1 expression (OR=0.665, 95%CI: 0.472-0.937), smoking index ≤400 (OR=1.72, 95%CI: 1.061-2.789) and ECOG=2 (OR=3.551, 95%CI: 2.133-5.914), extensive-stage (OR=2.487, 95%CI: 1.793-3.451) and SVCS due to lung cancer (OR=2.394, 95%CI: 1.49-3.846) were independent prognostic factors for SCLC patients.

**Conclusion:**

Prognosis of SCLC was related to TTF-1 expression independently after adjusting smoking, ECOG score, stage and SVCS due to lung cancer. Detection of TTF-1, CD56 and P40 expression level might be helpful for predict the prognosis of SCLC.

肺癌是我国以及世界范围内发病率和死亡率最高的肿瘤之一，其中小细胞肺癌占13%-15%，预后较差，侵袭性强，与吸烟状态相关^[1, 2]^。小细胞肺癌主要分为局限期和广泛期^[3]^。如果能在疾病较早期对小细胞肺癌预后有所判断，制定合理的治疗方案积极进行救治，达到改善生活质量，延长生存的目的。有研究显示，某些免疫表型标记物，如神经内分泌标志物、甲状腺转录因子1（thyroid transcription factor-1, TTF-1）、CD56、P40等在肺癌的发生发展中具有重要的作用，并应用于临床病理诊断。TTF-1是一种分子量为40 kDa的核蛋白，是NKx2转录基因家族的成员之一最常见其在甲状腺和肺组织中表达，并在甲状腺和肺组织癌变过程中起着重要的作用，调控多种基因的表达^[4]^。TTF-1为Ⅱ型肺泡细胞和Clara细胞的特异性标记物，在肺小细胞癌与肺泡细胞癌中表达率高，目前临床中常用于非小细胞肺癌病理分型诊断和鉴别诊断的免疫组化标志物有：Napsin A和TTF-1常用腺癌的诊断，CK5/6、p63以及p40常用于鳞状细胞癌的诊断。而这些指标在小细胞肺癌中的表达如何，特别是小细胞肺癌的预后中研究较少，部分研究的结果也并不一致。本研究通过198例小细胞肺癌患者及其癌组织标本的回顾性分析，探讨TTF-1、神经细胞粘附分子CD56、P40蛋白以及临床特征在小细胞肺癌预后中价值，以期为其他研究提供参考。

## 1 材料与方法

### 1.1 研究对象及资料搜集

收集2009年1月1日-2014年1月1日来首都医科大学宣武医院胸外科初次就诊并确诊的小细胞肺癌患者石蜡包埋组织标本及临床病理资料。患者纳入标准：①病例经肺穿刺、纤维支气管镜或手术病理标本确诊为小细胞肺癌；②石蜡包埋病例肺癌组织标本保存完好，有完整的临床病历资料；③年龄 > 18周岁；④接受以铂类为基础的一线药物联合化疗，治疗方案包括依托泊苷联合卡铂/顺铂（EP/CE），托泊替康联合卡铂/顺铂（IP/IC）；⑤患者有明确的随访资料，包括电话、地址等，依从性好。

收集患者的临床资料，包括采集病例相关的临床病理资料，包括年龄、性别、吸烟史、美国东部肿瘤协作组（Eastern Cooperative Oncology Group, ECOG）评分、病理类型、是否合并上腔静脉综合征及临床分期。分别根据第7版美国癌症联合委员会（American Joint Committee on Cancer, AJCC）国际肿瘤分期进行肿瘤-淋巴结-转移（tumor-node-metastasis, TNM）分期以及美国退伍军人肺癌研究组（Veterans Administration Lung Study Group, VALG）制定的分期进行局限期和广泛期划分；根据ECOG评分标准进行体力状态评分。总生存时间（overall survival, OS）定义为患者开始治疗至患者死亡或末次随诊时间。无疾病进展时间（progression free survival, PFS）为从第一次给药至疾病进展或死亡时间。末次随访时间为2017年4月。

### 1.2 实验方法及试剂

采用免疫组化EnVision两步法，进行TTF-1、P40、CD56抗体检测。按照4 μm左右厚度把癌组织切片，经过二甲苯中脱蜡、梯度酒精水化、PBS清洗、抗原修复后，滴加I抗，孵育，滴加增强复合物，滴加Ⅱ抗，置于湿盒内，37 oC孵育30 min，PBS清洗后，DAB试剂盒显色，苏木精复染细胞核，盐酸酒精分化，酒精脱水，二甲苯透明30 min，中性树胶1滴，盖波片封片。免疫组化试剂盒为北京中杉金桥生物技术有限公司产品。

### 1.3 结果判定

分别由两位有经验的病理科医生阅片审核。TTF-1、P40蛋白均定位于细胞核，呈棕黄色颗粒，以细胞核中出现棕黄色为阳性细胞。CD56着色部位均在细胞质或细胞膜，以细胞质或细胞核内出现背景清晰的黄色或棕黄色颗粒为阳性细胞。综合分析整张切片的阳性细胞数及着色强度：阳性细胞数 < 10%，着色较淡者判定为阴性（-）；阳性细胞数≥10%，可见棕黄色颗粒者判定为阳性（+）。

### 1.4 数据统计与分析

使用SPSS19.0统计包进行数据处理及分析。计量资料用均数±标准差（Mean±SD）表示，用*t*检验比较差别；计数资料用频数（*n*）和构成比（%）表示，用*χ*^2^检验等非参数方法比较差别。生存分析采用*Kaplan-Meier*法，生存率的比较采用*Log-rank*法进行显著性检验。使用单因素和多因素*Cox*比例风险回归模型分析生存时间和生存状态与因素之间的关系，其中，已经死亡和存活作为因变量，其他变量作为自变量，首先进行单因素分析，单因素分析有意义的变量进入下一步的多因素分析，多因素分析采用逐步回归法。*P* < 0.05为差异有统计学意义。

## 2 结果

### 2.1 患者临床资料及与TTF-1、CD5、P40表达基本情况

198例小细胞肺癌患者平均年龄（62.7±11.7）岁。其中接受胸部放疗者130例（65.7%），未接受者68例（34.3%）；接受依托泊苷联合卡铂或顺铂方案（CE/EP）化疗者161例（81.3%），托泊替康联合卡铂或顺铂方案（IC/IP）32例（16.2%），其他方案5例（2.5%）。截止最后一次随访，共有156例死亡，其他42例为截尾数据，死亡者平均存活天数为（588.4±498.6）d。TTF-1构成比在截尾和死亡的患者中有显著差异（*χ*^2^=6.007, *P*=0.014）（表 1）。

**1:** 198例小细胞肺癌患者基本情况及TTF-1、CD56、P40表达情况 Clinical characteristics and expression of TTF-1, CD56 and P40 in 198 SCLC patients

Index	*n* (%)	TTF-1 positive [*n* (%)]	CD56 positive [*n* (%)]	P40 positive [*n* (%)]
Age (yr)				
≥60	126(63.6)	89(61.4)	111 (63.4)	8 (57.1)
< 60	72 (36.4)	56 (38.6)	64 (36.6)	6 (42.9)
Gender				
Male	159(80.3)	119(82.1)	140(80)	10(71.4)
Female	39 (19.7)	26(17.9)	35 (20)	4 (28.6)
ECOG				
0	36 (18.2)	30 (20.7)	33 (18.9)	0 (0)
1	120 (60.6)	89(61.4)	105 (60.0)	9 (64.3)
2	42 (21.2)	26(17.9)	37(21.1)	5 (35.7)
Smoking index				
0	49 (24.7)	36 (24.8)	45 (25.7)	0 (0)
≤400	40 (20.2)	24(16.6)	33 (18.9)	3 (21.4)
> 400	109(55.1)	85 (58.6)	97 (55.4)	11 (78.6)
Stage				
Limited	114 (57.6)	83 (57.2)	99 (56.6)	6 (42.9)
Extensive	84 (42.4)	62 (42.8)	76 (43.4)	8 (57.1)
TNM stage				
Ⅱ	10 (5.0)	7 (4.8)	10 (5.7)	0 (0)
Ⅲ	76 (38.4)	55 (37.9)	97 (55.4)	6 (42.9)
Ⅳ	112 (56.6)	83 (57.3)	68 (38.9)	8 (57.1)
SVCS				
No	177 (89.4)	131 (90.3)	155 (88.6)	13 (92.9)
Yes	21 (10.6)	14 (9.7)	20(11.4)	1 (7.1)
Smoking index: number of cigarettes smoked per day x number of years of smoking. SVCS: superior vena cava syndrome; ECOG: Eastern Cooperative Oncology Group; TTF-1: thyroid transcription factor-1; SCLC: small cell lung cancer.				

### 2.2 病理学结果情况

免疫组化染色显示，198例小细胞肺癌患者TTF-1、CD56、P40的阳性率分别为73.2%（145/198）、88.4%（175/198）、7.1%（14/198）。镜下观察绝大部分小细胞肺癌癌组织细胞为小细胞，形态单一，胞质稀少，近乎裸核，核为圆形或者类圆形，染色质较为丰富且分布较为均匀，呈细颗粒状，核膜薄，核仁不明显；突出特点是大部分组织挤压较重，形成拉丝的染色质条纹，形态欠清（图 1）。

**1:**
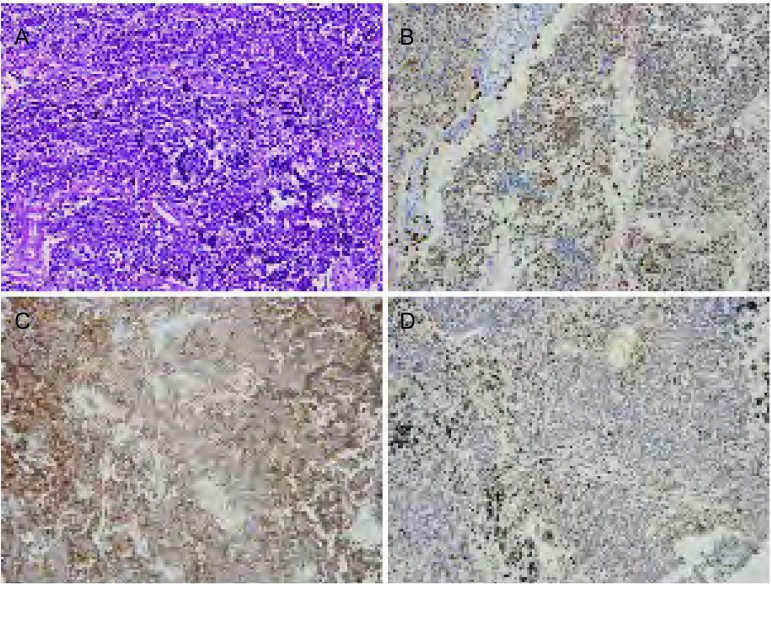
HE染色图片和部分免疫标记物免疫组化染色图片(×200)。A：HE染色；B：TTF-1阳性；C：CD56阳性；D：P40阳性。 HE staining and biomarker expression by immunohistochemistry (×200). A: HE staining; B: TTF-1 positive; C: CD56 positive; D: P40 positive.

### 2.3 单因素和多因素素逐步*Cox*风险比例模型分析

分别通过单因素和多因素逐步*Cox*风险比例模型分析，发现调整其他因素的影响之后，TTF-1是否阳性为小细胞肺癌患者独立预后因素OR=0.665，95%CI：0.472-0.937，*P*=0.006；CD56、P40表达情况与预后无相关性。其他与被调整的、有显著性的相关因素包括：与不吸烟者相比，吸烟指数≤400组OR=1.72，95%CI：1.061-2.789，*P*=0.015；与ECOG得分为0者相比，得分为2分的OR=3.551，95%CI：2.133-5.914，*P* < 0.001；疾病处于广泛期的较局限期的OR=2.487，95%CI：1.793-3.451，*P*=0.002；合并上腔静脉压迫综合征（superior vena cava syndrome, SVCS）者OR=2.394，95%CI：1.49-3.846，*P*=0.008（表 2）。

**2:** 小细胞肺癌预后单因素和多因素*Cox*风险比例模型分析 Univariate and multivariate *Cox* proportional hazards model analysis of OS for SCLC patients

Index	Comparison	Univariate			Multivariate	
		OR (95%CI)	*P*		OR (95%CI)	*P*
Gender	Male *vs* Female	0.722 (0.476-1.095)	0.125			
Age	≥60 yr *vs* < 60 yr	1.639 (1.175-2.285)	0.004			
Smoking index	≤400 *vs* 0	1.72 (1.061-2.789)	0.028		1.879 (1.13-3.125)	0.015
	> 400 *vs* 0	1.377 (0.917-2.069)	0.123			
ECOG	1 *vs* 0	1.196 (0.776-1.843)	0.417			
	2 *vs* 0	3.551 (2.133-5.914)	< 0.001		3.202 (1.852-5.539)	< 0.001
Stage	ED *vs* LD	2.487 (1.793-3.451)	< 0.001		1.735 (1.216-2.476)	0.002
TNM stage	Ⅲ *vs* Ⅱ	1.329 (0.538-3.281)	0.538			
	Ⅳ *vs* Ⅱ	3.308 (1.325-8.258)	0.010			
SVCS	Yes *vs* No	2.394 (1.49-3.846)	< 0.001		1.988 (1.201-3.291)	0.008
Chemotherapy	IC/IP *vs* CE/EP	0.956 (0.622-1.471)	0.838			
RT	Yes *vs* No	0.567 (0.408-0.788)	0.001			
TTF-1	Positive *vs* Negative	0.665 (0.472-0.937)	0.020		0.620 (0.439-0.875)	0.006
CD56	Positive *vs* Negative	1.124 (0.669-1.887)	0.660			
P40	Positive *vs* Negative	0.957 (0.644-1.422)	0.827			

### 2.4 生存曲线分析

TTF-1阴性者平均生存天数为（640.3±87.0）d，阳性者平均生存天数为（1, 063.0±117.5）d，两者的生存率情况有显著差异（*χ*^2^=5.516, *P*=0.019）（图 2）。

**2:**
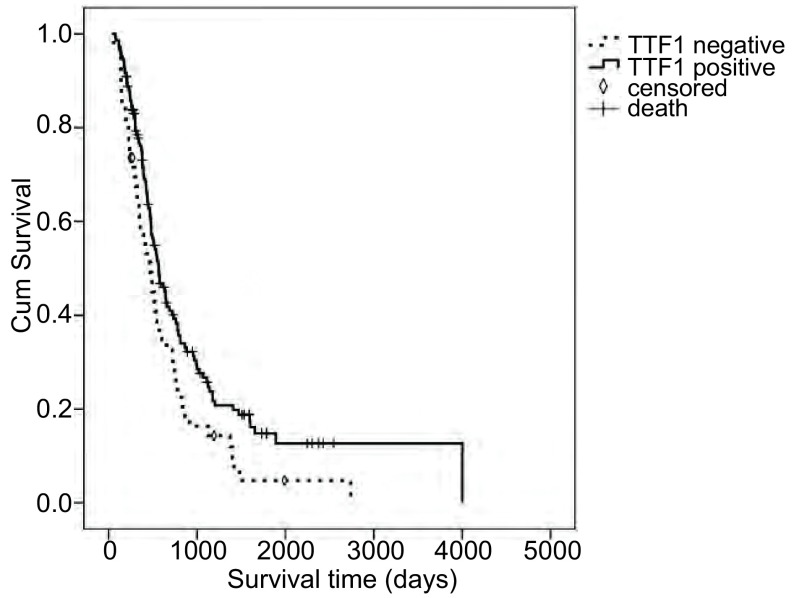
不同TTF-1表达的小细胞肺癌患者生存分析 *Kaplan-Meier* curves for SCLC patients according to TTF-1

## 3 讨论

本研究通过198例小细胞肺癌，分析癌组织的免疫表型标记物表达及其他患者临床特征与预后之间的关系。3个免疫组织化学标志物中，TTF-1、CD56处于较高表达水平，阳性比例超过70%，P40表达水平较低，阳性率仅为7.1%，与其他文献报道具有一致性。因此通过免疫表型标志物阳性情况可对小细胞肺癌的具有辅助诊断和鉴别诊断作用，当然诊断应结合其他小细胞肺癌的临床特征，最终确诊仍要靠病理。小细胞肺癌的预后与免疫表型标志物及临床情况等多个因素相关，TTF-1是否阳性为小细胞肺癌患者独立预后因素，表现为TTF-1阳性的患者生存期可能更长，其他两种标志物CD56、P40表达情况与预后没有相关。临床因素方面，与不吸烟者相比，吸烟、ECOG评分、分期是小细胞肺癌的独立相关因素，表现为吸烟者、ECOG评分较差、广泛期患者生存期更短。

TTF-1为甲状腺特异性增强子结合蛋白，作为一个核转录因子，主要在前脑、甲状腺上皮、胚胎肺泡上皮细胞以及正常成人气管及肺泡上皮细胞中，尤其是许多肺部肿瘤包括肺腺癌、小细胞肺癌等等^[5]^。TTF-1与癌症发生、分化以及转移过程密切相关，在肺癌的诊断和预后中较为重要，但作用机制不是很清楚。TTF-1是Ⅱ型肺泡上皮细胞的标志物，参与调节肺上皮组织发育、分化，阳性者可能表示癌组织尚具有部分正常分化能力，侵袭力较弱^[6, 7]^。有关TTF-1与小细胞肺癌预后的关系研究较少，结论也不一致。Kasmann等^[8]^对放射治疗的局限性小细胞肺癌的研究发现，TTF-1没有进入预后的多因素分析模型。Misch等^[9]^研究认为TTF-1与晚期小细胞肺癌患者预后没有显著相关，但可作为一线化疗反应的预测指标。关于TTF-1和肺癌预后的关系在非小细胞肺癌的研究中更加常见，但是研究结论也存在不一致，有研究发现，Martins等^[10]^通过51例肺腺癌观察认为，TTF-1可以作为肺腺癌患者独立的预后指标。Yaman等^[11]^通过80例非小细胞肺癌研究认为，TTF-1与患者的生存相关没有显著性。Tan等^[12]^随访观察认为，TTF-1阴性表达者非小细胞肺癌更具侵袭性，阳性表达者中位生存期明显超过阴性表达者，可以作为非小细胞肺癌的预后指标。

TTF-1表达在不同部位、不同组织、不同器官、不同类型肿瘤是存在差异的，如在肺腺癌中表达率较高，也有研究^[13]^认为与肿瘤的不同分期有关，在TNM分期越高的小细胞肺癌患者中，阳性率越低。虽然本研究认为TTF-1在小细胞肺癌组织的表达水平较高，阳性率为83%，与其他研究相似，但其表达与年龄、性别、分期等临床特征无关。高宗炜等^[14]^研究发现TTF-1 DNA结合活性在肺癌组织较高，肺腺癌组织TTF-1 DNA结合活性光密度值显著高于其他病理类型。有研究分析非肺来源的小细胞肺癌，认为其他部位的小细胞癌与肺小细胞癌具有不同的标志物表达谱。Ordonez^[15]^发现肺外小细胞癌中TTF-1表达阳性率仅为7.4%，显著低于小细胞肺癌的96%。Yao等^[16]^对前列腺小细胞癌研究发现，TTF-1阳性率高达83%。因此TTF-1不是某个组织来源的特异性标志物，而与肿瘤细胞来源有关，这主要是神经内分泌来源的肿瘤细胞。通过分析非肺来源的小细胞癌以及小细胞肺癌中其他神经内分泌来源标志物也同时可以印证这一点，比如CD56在本研究中也有较高阳性率。

总之，本研究认为小细胞肺癌中的预后与TTF-1表达及吸烟、ECOG得分、癌瘤分期、合并SVCS等多个因素相关。TTF-1、CD56、P40等免疫表型标志物在部分小细胞肺癌病理组织中呈现阳性表达，对小细胞肺癌的诊断和鉴别具有辅助作用。但是本研究为单中心、回顾性研究，有一定的局限性，未来还需要前瞻性、大样本、多中心研究进行验证。
